# 533 Medical Photo Application–Delivering Expert Burn Care in the Intermountain West

**DOI:** 10.1093/jbcr/irad045.130

**Published:** 2023-05-15

**Authors:** Elizabeth Mark, Zach Rizzi, Giavonni Lewis

**Affiliations:** University of Utah Health, Salt Lake City, Utah; University of Utah Health, Salt Lake City, Utah; University of Utah Health, Salt Lake City, Utah; University of Utah Health, Salt Lake City, Utah; University of Utah Health, Salt Lake City, Utah; University of Utah Health, Salt Lake City, Utah

## Abstract

**Introduction:**

As the only verified Burn Center in the Intermountain West, we are faced with the challenge of providing care and support to a referral area spanning five states. In an effort to make consulting with our Burn Center more efficient and direct, we provide TeleHealth services. We typically provide real-time facility-to-facility consultation, facility-to-patient consultation, phone consultation, and photo consultation. Prior to development of our medical photo application (MedPic app), photo consults were completed via encrypted email. The consult process was inefficient resulting in multiple phone calls for one consult. As a result, we received requests to improve the process, making it more user friendly and HIPPA compliant.

Initially, our Burn Center created a photo consultation website that enabled referring providers to go to a webpage and upload photos. The consult began with a phone call from the referring provider. The Burn Center staff would inform the referring provider of the photo website and the referring provider would upload the photos. This improved the photo consult process, however it was still inefficient.

Based on these requests from our referring providers, staff, and patients, we developed the MedPic app. With the new MedPic app, we provide an easy to access and efficient process to consult with our Burn center; thus improving efficiency, reducing unnecessary hospital admissions, and associated medical transport costs.

**Methods:**

The photo app was created and made available in March of 2020. The app streamlined the consult process by creating a direct link from referring providers to Burn providers. Burn providers receive images as soon as they are uploaded through the app.

The MedPic app is a photo consultation tool and requires three steps: 1. Take photos of the wounds; 2. Enter patient information; and 3. Enter a return call back number. A plan of care is communicated back to the referring provider within 30 minutes. Photos taken within the app are not saved to any device. The images are encrypted to protect patient information making the app HIPPA compliant.

We collected encounter data generated by photo consultation to our burn center prior to and after the MedPic app development (January 2016- September 2022).

**Results:**

Since the MedPic app was released in March of 2020 we have noted 2,071 app downloads and have seen a steady increase in photo consultations. Figure 1 shows the total number of photo consultations to our Burn Center from January 2016 through September 2022.

**Conclusions:**

Our Burn Center created the MedPic app resulting in over 2,000 downloads. The app is integrated into our Burn Center Telehealth consultation workflow. By providing a direct approach to acute burn injury consultations, we are able to efficiently assess patient wounds and ensure the patient receives the level of care they need in a timely manner.

**Applicability of Research to Practice:**

This directly impacts triage and management of outpatient acute burn injuries.

